# Prevalence and concordance of high cardiovascular disease scores in HIV/AIDS patients from Croatia and Serbia with four international algorithms

**DOI:** 10.7448/IAS.17.4.19549

**Published:** 2014-11-02

**Authors:** Josip Begovac, Gordana Dragovic, Klaudija Viskovic, Jovana Kusic, Marta Perovic Mihanovic, Davorka Lukas, Djordje Jevtovic

**Affiliations:** 1 Department of Infectious Diseases, University Hospital for Infectious Diseases, Zagreb, Croatia; 2 Department of Pharmacology and Clinical Pharmacology, School of Medicine, University of Belgrade, Belgrade, Serbia and Montenegro; 3 HIV/AIDS Unit, Institute for Infectious and Tropical Diseases, Belgrade, Serbia and Montenegro

## Abstract

**Introduction:**

We evaluated cardiovascular risks in HIV-infected patients from Croatia and Serbia and the eligibility for statin therapy as recommended by the 2013 American College of Cardiology/American Heart Association (ACC/AHA) guidelines, European AIDS Clinical Society (EACS) Guidelines and European Society of Cardiology and the European Atherosclerosis Society (ESC/EAS) guidelines for cardiovascular disease (CVD) prevention [1–3].

**Materials and Methods:**

A cross-sectional analysis of consecutive patients between 40 and 79 years old who had received antiretroviral therapy for at least 12 months was performed.

**Results:**

Of 254 (132 from Croatia and 122 from Serbia) persons included in the study, 76% were male; median age was 49 years. Up to 51.6% of persons had a high CVD risk. The prevalence of current smoking was 42.9%, hypertension 31.5% and hypercholesterolaemia (>6.2 mmol/L) 35.4%. Statins would be recommended to 21.3% (95% CI, 16.3% to 27.4%) of persons by the EACS, 25.6% (95% CI, 20.2% to 31.9%) by ESC/EAS and 37.9% (95% CI, 31.6 to 44.6%) by the ACC/AHA guidelines. A high 5-year data collection on adverse effects of anti-HIV drugs study risk score (>5%) had a moderate agreement with the high (≥20%) 10-year CVD Framingham risk score (kappa=0.47) and high (≥5%) 10-year European systematic coronary risk evaluation score algorithm (kappa=0.47), and substantial agreement with the elevated (≥7.5%) 10-year Pooled Cohort Atherosclerotic CVD risk equation score (kappa=0.63).

**Conclusion:**

We found a high prevalence of CVD risks in patients from Croatia and Serbia. The ACC/AHA guideline would recommend statins more often than ESC/EAS and EACS guidelines.
